# 

**Published:** 1921-07-23

**Authors:** 


July 23, 1921] [Price Twopence

### The Hospital

The Workers' Newspaper of Administrative Medicine and Institutional Life, Administration, National Insurance and Health.

CONTENTS

**The King's Fund and the Government Grant** ... 273

**The Voluntary Hospitals Commission** ... ... 274

Official Policy Defined.

**Notes of the Week** ... ... ... ... ... .. 275
The B.M.A. at Newcastle—Medical Secrecy—The Mischief in Cocktails—A Financial Recovery at Norwich—The Hospital Pageant—A Result of Mass Contributions—A Royal Visit—A Five Years' Record—An Index of Cancer Cures.

**The College of Surgeons Mustum** ... ... 27

Acquisitions and Research.

**The Use and Abuse of Medical Literature** ... 278

**The Society of Radiographers** ... ... ... 278
Competition Thesis in Radiography and Electro-Therapy.

**Guy's Hospital War Memorial** ... ... ... 279

Unveiling by the Duke of York.

**The Financing of the London Hospitals** ... ... 280
A Still Greater Effort Required.

**The Oxford Scheme** ... ... ... 281

As Organised by and Worked at the Radcliffe Infirmary.

**A Note on Scottish Hospital History** ... ... 282

**The Public Well-Being** ... ... ... ... ... ... 283

Sir George Newman's Annual Report.

**New Health Legislation** ... ... ... ... 284
Powers Obtained by Grimsby Corporation.

**The Editor's Letter-Box** ... ... ... ... 284

Doctor and Patient.

**The Matrons' and Sisters' Department** ... ... 285

The Non-Residential Preliminary School.

**Round the Hospitals** ... ... ... ... 285

**Investiture by the King** ... ... ... ... 287

**Presentation to Miss Hoadley** ... ... 287

**The General Nursing Council of England and Wales** ... ... ... ... ... ... ... ... 288

**Parliament and the Professions** ... ... ... 289

**The College of Nursing (Limited by Guarantee)** 290

**Forthcoming Events** ... ... ... ... ... ... 290

